# Charge Mediated Changes to the Intrinsic Viscosity of Biopolymer Systems

**DOI:** 10.3390/polym16202894

**Published:** 2024-10-14

**Authors:** Anand Raja, Philipp K. Wilfert, Stephen J. Picken

**Affiliations:** 1 Advanced Soft Matter, Department of Chemical Engineering, Faculty of Applied Sciences, Delft University of Technology, van der Maasweg 9, 2629 HZ Delft, The Netherlands; s.j.picken@tudelft.nl; 2 Environmental Biotechnology, Department of Biotechnology, Faculty of Applied Sciences, Delft University of Technology, van der Maasweg 9, 2629 HZ Delft, The Netherlands; p.k.wilfert@tudelft.nl

**Keywords:** biopolymers, coil size, intrinsic viscosity, persistence length, molar mass

## Abstract

A theoretical approach is presented to quantify the effect of ionic strength on the swelling and shrinkage of the hydrodynamic coil size of a generic biopolymer. This was conducted in view of extraction methods that often utilize acids and alkali combinations and, therefore, invariably impact the levels of salt found in commercially available biopolymers. This approach is supplemented by intrinsic viscosity measurements for the purpose of validation across a variety of biopolymer architectures, type of functionalization, as well as the quoted molar mass. By accurately capturing the magnitude of change in the coil size, it is discussed how a biopolymer coil size is far more sensitive to changes in the ionic strength than it is to the molar mass (or contour length) itself. In turn, it is highlighted why the current characterization strategies that make use of weight-averaged molar mass are prone to errors and cannot be used to establish structure—property relationships for biopolymers. As an alternative, the scope of developing an accurate understanding of coil sizes due to changes in the “soft” interactions is proposed, and it is recommended to use the coil size itself to highlight the underlying structure—property relationships.

## 1. Introduction

From a generic point of view, most biopolymers may be described as polymers that are decorated by functional groups [1]. These groups exhibit thermodynamically favorable “soft” interactions and may, therefore, present variations in their levels of protonation due to changes in the pH [2,3]. Moreover, screening effects due to changes in the ionic strength and favorable bridging (via physical crosslinks) based either on the composition of counter ions in solution or intramolecular interactions such as H-bonding with other functional groups are critically important [1,2,3]. Certainly, in some cases, the specific effects of these interactions on both the structure as well as the properties have been studied in more detail. One such recent and widely cited example includes the prediction of the folded conformation of proteins through the knowledge of their polypeptide sequence [4].

However, the specificity of such approaches largely overlooks the general trends that may be observed in the structure and properties of all biopolymers, which occur due to changes in the pH or ionic strength. Although there are examples that adopt such a generic methodology [5,6], the effort to develop such approaches further remains absent. Thus, there is sufficient scope to extend the generic physical understanding on the structure and properties of a biopolymer. Based on the methods outlined by Picout et al. [6], a choice was made to extend the rheological understanding of biopolymers further, and thus, an attempt to map changes in the hydrodynamic coil size of a generic biopolymer is presented below.

**Figure 1:**
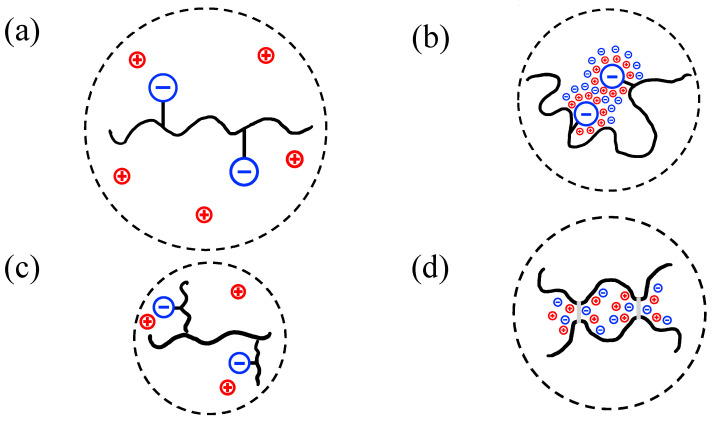
Expected coil conformations for different conditions: (**a**) unscreened linear biopolymer coil, (**b**) screened linear biopolymer coil, (**c**) unscreened branched biopolymer coil with the same molar mass as the linear coil, and (**d**) screened biopolymer coil that forms physical crosslinks. The dashed lines schematically represent the hydrodynamic (pervaded) volumes occupied by the coils.

In Figure 1, a schematic map of some expectable differences in coil sizes by varying the pH and ionic strength is shown. However, these schematics merely present a coarse-grained physical interpretation of the coil size. What is lacking, then, is a theoretical and quantitative measure for changes in the coil size due to functional group interactions. However, it is clearly not possible to map the effect of all these changes at once. Thus, this study is limited to predominantly cover the effects of ionic strength on the hydrodynamic size of charged biopolymer coils.

As such, substantial ionic strength levels are to be anticipated in biopolymer systems in view of the extraction (or isolation) methods that utilize techniques such as alkaline dissolution followed by acid precipitation (or vice versa) [2,7]. The large changes in the pH that are used invariably introduce salts within the system and substantially increase the ionic strength. Further, downstream processing steps may be employed to remove the excessive levels of salts [7] in order to meet the yields and purities expected, and these steps, therefore, are instrumental in reducing the ionic strength. Moreover, for the application of such biopolymer systems, the rheological processing behavior and, indeed, the final properties will critically depend on the ionic strength of the system. Thus, for the purposes of extraction, processing, and applicability of biopolymers, it is important to establish the sensitivity to variations in ionic strength within these systems.

Before highlighting the theoretical approach, a brief outlook on the choice of a measure for the quantitative coil size is provided. Whilst the size of coils may be calculated conveniently using size exclusion chromatography (SEC) or light scattering, the intention was to map the coil sizes using intrinsic viscosity. As it was impractical to account for a variety of standards representative of biopolymer systems under consideration [8], it would have been necessary to deal with inaccuracies in the SEC results depending on the level of screening. Equally, the inaccuracies associated with curve-fitting protocols in commercially viable light-scattering techniques were taken into consideration [9]. In contrast, it has been established that the (intrinsic) viscosity may be precisely measured across a wide variety of polymer systems [10].

As elucidated by Lopez and coworkers in their extensive review [11], the modelling of polyelectrolytes (in this case, biopolymers as weak polyelectrolytes) using worm-like chains allows for the estimation of the intrinsic viscosity and, thus, serves as the basis of the theoretical approach. This is covered extensively in Section 2. In Section 3, the list of biopolymers and methodology are presented. The results are reported in Section 4, and the major findings and drawbacks of estimating a biopolymer’s molar mass using the size (or length) of coils are highlighted in Section 5. Finally, in Section 6, a general outlook for future work in this direction is provided.

## 2. Theoretical Review

The intrinsic viscosity may be calculated using the Einstein equation for very dilute polymer systems, where they are traditionally known to behave as a Newtonian fluid [10]:

(1) $$\eta =\eta_{s}\left(1+2.5\phi \right)=\eta_{s}\left(1+\left[\eta \right]c\right)$$

Here, η represents the viscosity of the bulk system, $\eta_{s}$ represents the solvent viscosity, ϕ represents the volume fraction of polymer coils, $\left[\eta \right]$ represents the intrinsic viscosity of the polymer coils, and c represents the (mass) concentration of the polymer in solution. The intrinsic viscosity is typically represented in the inverse units of mass concentration and, therefore, should be recognized as a measure of the coil mass density or, indeed, the macromolecular mass density if the architecture is more complex than a linear chain. Further, as discussed by Rubinstein and Colby [10], the end-to-end distance of a (sufficiently long) worm-like chain may be approximated using the following relation:

(2) $$\langle R^{2}\rangle \approx 2L_{p}L_{c}$$

Here, 〈R〉 is the (average) end-to-end distance, and $L_{p}$ is the persistence length of the chain. $L_{c}$ in Equation (2) is the contour length of the chain and may be calculated by taking the product of the number of repeating units (N) and the single monomer projected length (l). As discussed by Lopez [12], Norisuye and coworkers have provided extensive experimental evidence to show that the radius of gyration of polyelectrolytes in the excess salt limit can be described using the worm-like chain model. Thus, the end-to-end distance may then be used to calculate the radius of gyration [10]:

(3) $$\langle R_{g}^{2}\rangle =\frac{\langle R^{2}\rangle }{C}$$

Here, $\langle R_{g}\rangle$ is the radius of gyration. C in Equation (3) is an integer whose value depends on the persistence length. For example, in the case of linear chains, C=6 for an ideal chain (coil limit), whereas C=12 for a rod-like chain. In a simplified approach, the Fox–Flory equation [10] can be used to calculate the intrinsic viscosity:

(4) $$\left[\eta \right]\approx \frac{\langle R_{g}^{3}\rangle }{M}$$

where M is the molar mass of the polymer chain. However, no particular attention is paid to the hydrodynamics of the worm-like cylindrical chain, as the radius of gyration (in place of the hydrodynamic radius) is used, as is, to calculate the intrinsic viscosity. Yamakawa and Fujii [13] specifically account for this by using the Oseen–Burgers procedure and make an approximation for the value of the intrinsic viscosity. However, in their effort to do so, they only provide analytical solutions for the rod limit as well as the coil limit. For all intermediate conformations, they only provide a numerical (or approximate) solution that is dependent on the length of the stretched-out chain (contour length):

(5) $$\left[\eta \right]\approx \phi_{YF}\frac{L_{c}^{3/2}}{M}$$

$\phi_{YF}$ in Equation (5) is a function [m^3/2^] whose value depends on the contour length. Although well intended, the solution provided by Yamakawa and Fujii reduces the subtle details about the stiffness of chains at relevant intermediated conformations using the semi-empirically calculated values of $\phi_{YF}$. Additionally, this model does not provide a means of estimating the intrinsic viscosity at various ionic strengths.

Another drawback of the Fox–Flory approach is the use of molar mass. As highlighted in Section 3, this information may not be readily available from the manufacturer for all biopolymer systems. Additionally, the molar mass of commercially available (bio)polymers is typically reported using weight-averaged molar mass [14] ($M_{w}$). As $M_{w}$ detemination is dependent on the size (or length) of the polymer coils, variations may be expected in its value depending on the pH and ionic strength of the solution. It is also worth noting that the current study is limited to biopolymer systems whose chemical structure is (somewhat) well defined. However, it is expectable that the chemical structure of the biopolymer system under consideration is not well defined at all [2,7], and thus, the molar mass as such may not be readily ascertained. Therefore, changes in the intrinsic viscosity may instead be relayed using changes in the persistence lengths in the screened vs. unscreened case. By doing so, it is equally possible to address the limitations surrounding the Yamakawa and Fujii approach.

As discussed by Dobrynin [15], Odijk, Skolnick, and Fixman (OSF) introduced the concept of the electrostatic persistence length for (semi) stiff polyelectrolyte chains. According to their approach, the persistence length of a polymer backbone may be written as the sum of the bare persistence length (referred to above as $L_{p}$) and the electrostatic persistence length ($L_{p}^{OSF}$):

(6) $$L_{p}^{+}=L_{p}+L_{p}^{OSF}\approx L_{p}+\frac{L_{B}f^{2}}{4{\left(\kappa l\right)}^{2}}$$

Here, $L_{B}$ represents the Bjerrum length, f represents the fraction of monomers that are charged, and $\kappa^{-1}$ represents the Debye length. By virtue of measuring the conductivity of the dilute unscreened polymer solutions, the concentration of NaCl in solution is estimated to be ~1 mM (see Section 3). This value can be used to calculate the value of $\kappa^{-1}$ (9.621 nm). Equally, it is possible to estimate the $L_{B}$ value of water at 298 K (0.714 nm). Therefore, the unscreened persistence length value may be calculated for different systems using Equation (6).

From Equations (2) and (3), it is clear that $\langle R_{g}\rangle \approx \;\langle R\rangle \approx \sqrt{L_{p}}$. Thus, upon substituting for $\langle R_{g}^{3}\rangle$ in Equation (4), it is clear that $\left[\eta \right]\approx L_{p}^{3/2}$. It further follows from Equation (6) that the ratio of the intrinsic viscosity in the unscreened case (subscript U) to that in the completely screened case (subscript S) may in term be represented as ${\left[\eta \right]}_{U}/{\left[\eta \right]}_{S}\approx {\left(L_{p}^{+}/L_{p}\;\right)}^{3/2}$. By substituting the full expression for $L_{p}^{+}$ from Equation (6), the following relation is obtained:

(7) $${\left[\eta \right]}_{R}=\frac{{\left[\eta \right]}_{U}}{{\left[\eta \right]}_{S}}\approx {\left(\frac{L_{p}^{+}}{L_{p}}\right)}^{\frac{3}{2}}={\left(\;\frac{L_{p}+L_{p}^{OSF}\;}{L_{p}}\right)}^{\frac{3}{2}}={\left(1+\frac{L_{p}^{OSF}}{L_{p}}\right)}^{\frac{3}{2}}={\left(1+\Theta_{p}\right)}^{\frac{3}{2}}$$

Here, ${\left[\eta \right]}_{R}$ is termed as the “relative intrinsic viscosity” and represents the ratio of the intrinsic viscosities (unscreened to screened). The term $\Theta_{p}$ represents the expansion to the bare persistence length and is, therefore, labelled as the “expansion factor”. Thus, the experimentally obtained relative intrinsic viscosity values are compared to the ones presented theoretically using Equation (7). The study is limited to polysaccharides and polypeptides, and Equation (7) is, therefore, employed for these two cases.

Lopez reports that there is some contention about the bare persistence length ($L_{p}$) of cellulosic backbones [12]. This may be attributed to the fact that cellulose itself remains insoluble in water, and so, its persistence length may only be calculated via soluble (or charged) derivates [16], where the charged systems additionally will have multiple f values. Thus, the uncertainty surrounding the exact value for the bare persistence length ($L_{p}$) of polysaccharides is recognizable. Calculations are, therefore, performed using multiple values. Equally, the fraction of charged monomers (f) is largely governed by the degree of substitution of the sugar rings. In specific cases, such as alginate or pectin, a further reduction in the f value may be expected. In the case of alginate, this is attributable to intramolecular H-bonding effects within the guluronic blocks [17], and in the case of pectin, this may be attributed to the methylation of the galacturonic acid fractions [2]. Thus, the f values were carefully selected for each polysaccharide system. Finally, there may be potential differences in monomer size (l) amongst different sections of the chain in cases such as alginate [18]. However, for the purposes of the calculations, a single value of monomer size [19] (l = 1 nm) is retained.

Unlike polysaccharides, polypeptides are mostly polyampholytes and, thus, show a reversal in swelling phenomena in the screened vs. unscreened cases. Not only this, in the unscreened and fully charged case (+ and −), the oppositely charged moieties have a greater affinity to each other and could, thus, lead to coil contraction or even collapse, frequently leading to the characteristic secondary structures, such as “folding” and “helix” formation, within the coils. Additionally, the f value is far more variable in the case of polypeptides. This is because the fraction of charged monomers is governed by the amino acid fractions that possess a charged side group and is, therefore, unique to each polypeptide sequence. This f value is further variable due to protonation of the different functional groups at different pH ranges (e.g., carboxylic groups vs. amine groups). Thus, it is not very easy to compare the swelling of multiple polypeptides directly with the theoretical approach that is highlighted, unless there is clear a priori knowledge of the chain conformation. Nevertheless, it is worth analyzing the theoretical calculations for an idealized (purely) anionic/cationic polypeptide and comparing it to polysaccharides. Such a system may be found under the correct levels of screening and/or (elevated) temperature [7]. Equally, it is worthwhile to compare experimentally obtained pH-associated coil swelling/compaction for fully screened polypeptides to screening-associated coil swelling/compaction in the case of polypeptides. Thus, theoretical calculations are presented for different f values in the case of polypeptides.

## 3. Materials and Methods

### 3.1. Materials

All the biopolymers used in this study were procured from Sigma—Aldrich (Zwijndrecht, The Netherlands). The list of polymers that were chosen is tabulated in Table 1 along with the information provided by the supplier and their salient features. The specific choice of biopolymers allows for us to compare the results for polyanionic, polycationic, as well as polyampholytic polymers. It was equally possible to compare the influence of salient chemical features such as blocks and branching. Additionally, there is also considerable variation in the molar mass (at least in cases where it is documented) across all biopolymers listed.

All biopolymer systems were prepared by stirring a desired concentration of the polymer in deionized water for a period of 24 h (86,400 s) in a sealed conical flask at 293 K. When not in use, the samples were stored in a refrigerator at 277 K to prevent degradation. The conductivity was increased, in the case of the screened samples, by adding the desired amounts (0.2–0.3 M) of NaCl to each individual biopolymer system. The pH changes within the gelatin sample were achieved using 1M HCl and 1M NaOH solutions in deionized water. Finally, the desired dilutions of the individual biopolymer systems were made using deionized water, whilst an effort was made to maintain the desired pH and ionic strength. All values of conductivity were measured at a temperature of 293 K and 50% RH in the lab environment.

**Figure 2:**
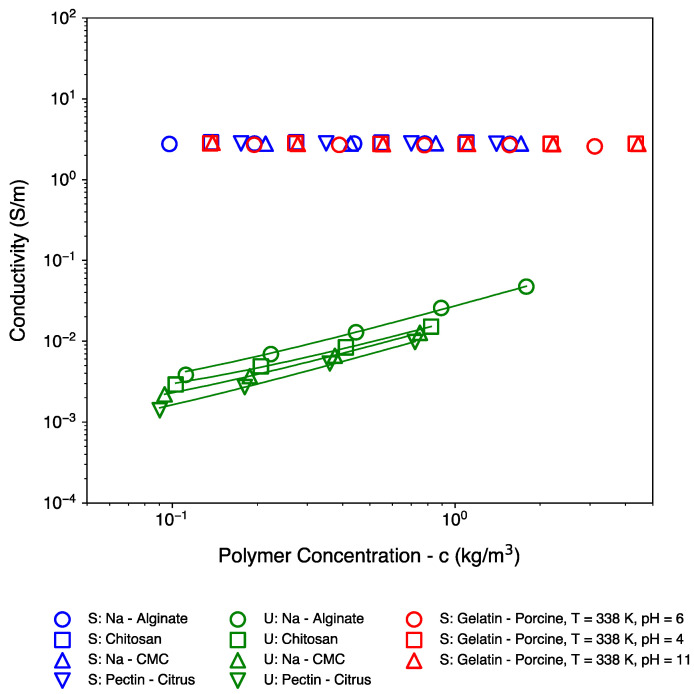
Conductivity of the biopolymer systems studied. S = screened, U = unscreened. Guides to the eye are also provided for the unscreened samples to highlight the differences in the values of their conductivity as well as the (almost) linear dependence with respect to concentration.

Figure 2 shows the conductivity of the samples. In view of the extraction methods for biopolymers, such as dissolution followed by precipitation, the conductivity of the unscreened samples could correspond to residual (surplus) salts that may be present within the samples. Alternatively, the measured conductivity values for some datasets are also in line with the expected values based on the counterion concentration along the polymer backbone [20,21] (see Supplementary Material Section S1). Regardless, as noticeable, upon subsequent dilution from higher concentrations, the conductivity dropped in a predictable and almost linear fashion. Thus, the polymer concentrations being represented on the x-axis of all Figures are believed to be within a relative error margin of 11% or 0.0453 decades.

### 3.2. Methods

The dilute biopolymer systems were tested for their intrinsic viscosity using a stress-controlled TA Instruments Discovery Hybrid Rheometer 3 (DHR-3, TA Instruments-Waters, Etten-Leur, The Netherlands). Due to the low polymer concentration and, therefore, viscosity, all measurements were performed using a concentric cylinder setup. A stainless-steel cup of diameter 30.36 mm and a stainless-steel DIN bob rotor with a diameter of 28.00 mm and a height of 42.07 mm were used. The DIN bob was maintained at a height of 5917.1 µm from the bottom surface of the cup.

All tests were performed at a temperature of 298 K except in the case of the gelatin samples, where the temperature was increased to 338 K to break the secondary and tertiary structure interactions within the polypeptide chain [22]. As the solutions behaved as Newtonian fluids, measurements at a suitable stress value (≤0.1 Pa) were sufficient. Because of the low viscosity, a low stress value was chosen to mitigate flow instabilities resulting from high shear rates. The samples were held under these conditions for a period of 600 s to ensure the development of the plateau value in the viscosity, free from any inertial effects. This setup was also used to obtain the reference viscosity of deionized/saline water under the following conditions: at 298 K with no added NaCl, at 298 K with added NaCl, and at 338 K with added NaCl.

## 4. Results

**Figure 3:**
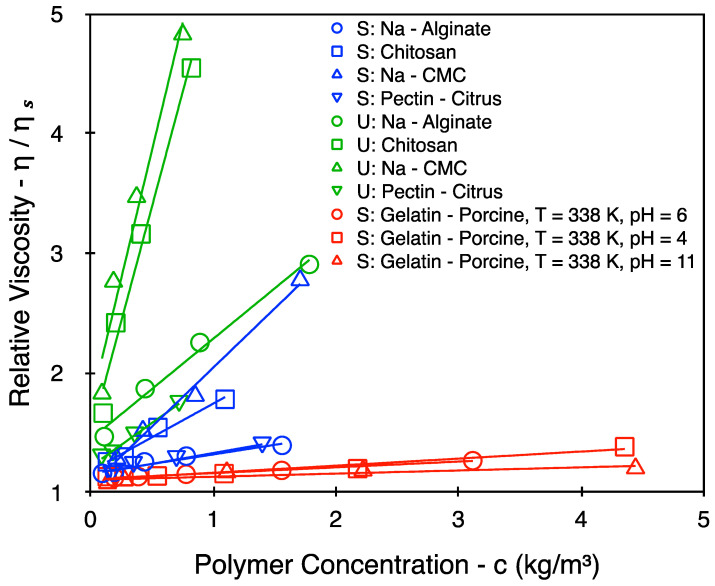
Experimentally obtained relative viscosity as a function of polymer concentration. S = screened, U = unscreened. These curves were subsequently used to calculate the intrinsic viscosity values from Equation (1). The straight lines act as guides to the eye to point out the linear slopes of each dataset.

Figure 3 represents the experimental datasets that were used to calculate the intrinsic viscosity. The y-axis of Figure 3 was obtained by dividing a particular system’s viscosity with that of the corresponding solvent. As elucidated earlier, these solvent viscosities were also experimentally obtained and are tabulated in Table 2. The intrinsic viscosity of all datasets in Figure 3 is calculated using Equation (1). These are tabulated individually for each dataset in Table 3 (conversion to the frequently used dL/g may be obtained upon multiplying all values by 10).

It is noticeable from Table 3 (as well as Figure 3) that, across multiple biopolymer systems, the intrinsic viscosity spans roughly two orders of magnitude: from 0.06 m^3^/kg in the case of gelatin (pH = 11 with screening) up to 5.62 m^3^/kg in the case of unscreened CMC. As expected, the transition from the screened to unscreened state (in the case of polysaccharides) is accompanied by an increase in the intrinsic viscosity (green datasets vs. blue datasets). Large differences in the value of the intrinsic viscosity may also be observed across various polysaccharide systems, irrespective of screening effects. For instance, the intrinsic viscosities of CMC and chitosan are significantly larger than the intrinsic viscosities of alginate and pectin to suggest an overall larger coil size in the former cases. In contrast, gelatin presents a much smaller intrinsic viscosity value overall, with very limited changes in its value across multiple gelatin systems. This is roughly in line with what one might expect for a more flexible polypeptide backbone.

The values from Table 3 are further used to calculate the relative intrinsic viscosity (${\left[\eta \right]}_{R}$) and compare to the theoretical models. To facilitate this comparison, both sets of values are tabulated against each polysaccharide in Table 4. The results for gelatin are tabulated separately in Table 5.

## 5. Discussion

### 5.1. Theory vs. Experiments

**Figure 4:**
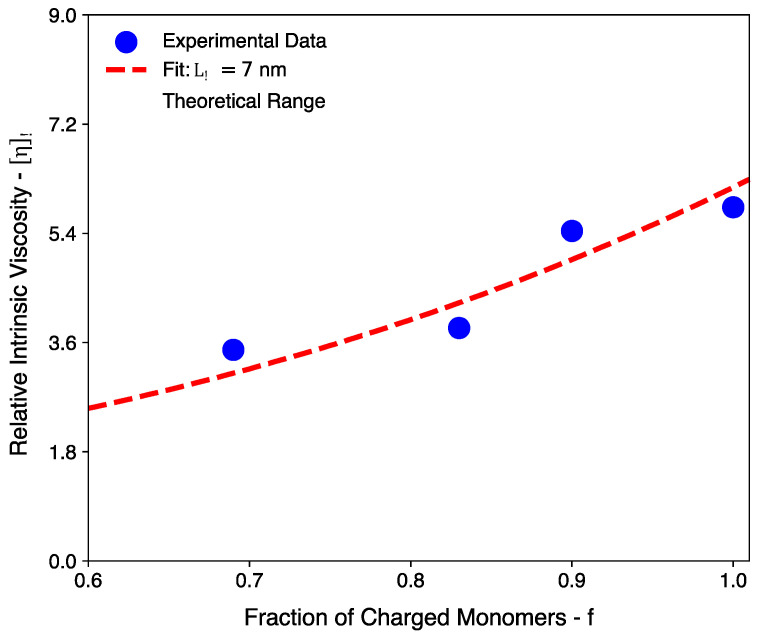
The dependence between the relative intrinsic viscosity and the fraction of charged monomers for polysaccharides. The data points represent the experimentally measured values for different polysaccharide systems (see Table 4). The shaded region corresponds to the theoretically estimated range that is calculated using $L_{p}$ values from 5 nm up to 10 nm (see Equation (7)). The dashed red line represents the optimal $L_{p}$ value (7 nm) used to fit the experimental results.

As highlighted in Section 2, there appears to be some uncertainty surrounding the exact value of the bare persistence length of cellulosic backbones. Thus, the theoretical results in Table 4 are presented for $L_{p}$ values from 5 to 10 nm. Lopez’s argument [12] that $L_{p}$ < 10 nm is supported by the rheological measurements presented here. Despite the somewhat large uncertainty, it is worth remarking that a fit for the experimental data is possible using an $L_{p}$ value of 7 nm (see Figure 4) and that this value is comparable with Lopez’s proposed range from 5 to 6 nm.

A clarification for the f values presented in Table 4 is also provided. To begin with, an f value of 0.69 is used for pectin based on the information provided by the supplier for the galacturonic acid content and the degree of methylation (74% *w*/*w* and 6.7% *w*/*w*, respectively, yielding ≈ 69%). Similarly, an average f value of 0.83 is used for sodium alginate based on the guluronic acid content found in multiple commercially available sodium alginates [23] (reported range between 0.80 and 0.86). In the case of sodium carboxymethyl cellulose, an f value of 0.9 is used, which corresponds to the degree of substitution stated by the supplier. Finally, for the chitosan system, an f value of 1.0 is used by virtue of every sugar ring possessing an amine group. Based on the range of $L_{p}$ values stated earlier and the range of f values, it is possible to gauge an expectation for the value of ${\left[\eta \right]}_{R}$ (Figure 4). Thus, the quantitative agreement between the OSF model and the experimental results is largely satisfactory (see the Supplementary Material Section S2 for the scaling approach [24]).

**Figure 5:**
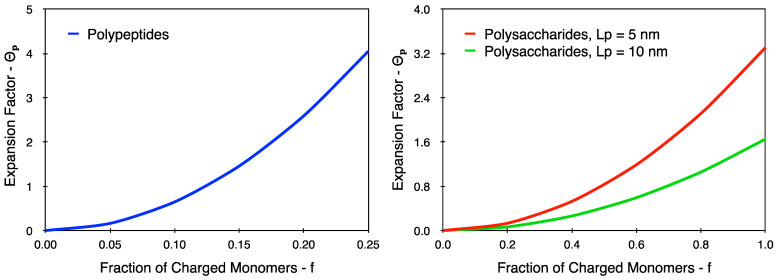
The dependence between the expansion factor ($\Theta_{p}$) and the fraction of charged monomers (f) for both polypeptides (**left**) and polysaccharides (**right**). The curves were obtained from Equations (6) and (7) for both cases.

The discussion for polypeptides begins with a clarification of the values presented in Table 5. The theoretical calculation for polypeptides is performed using a monomer length (l) of 0.371 nm. This is based on estimations for the polypeptide bond length by Corey and Pauling [25]. Additionally, an $L_{p}$ value of 1.855 nm is used for the theoretical calculations (based on the common approximation [26] that $L_{p}\approx$ 5l). From the experimental results, it is observable that there is a minimal shrinkage/swelling to the coil size of gelatin upon changing the pH. As discussed in Section 2, screening via salt addition within a polyampholyte system leads to an overall swelling within the system. In the case of the gelatin system, the polymer coils are further denatured by heating the system to 338 K. Thus, not only does it lose its ability to refold, it also is expectedly invariant to changes in the pH (or overall charge), thereby leading to very little variation in intrinsic viscosity (Figure 3 and Table 5). This hypothesis is further confirmed upon comparing the experimental results to the theoretical estimations for screening-related coil expansion in Table 5. It is observed that the pH-related changes in the screened system are comparable with the changes in coil conformations for a weakly charged polypeptide chain (f= 0.05 to 0.10). However, upon increasing the value of f to 0.25, there is a dramatic increase in the value of $\theta_{p}$ by virtue of a quadratic dependence between $\Theta_{p}$ and f (Equations (6) and (7)). This dependence is shown in Figure 5 for both cases, i.e., polysaccharides and polypeptides.

It is clear from Figure 5 that polypeptides exhibit a more marked quadratic dependence compared to polysaccharides. This is attributable to the fact that the size of a saccharide monomer is approximately 2.7 times larger than the size of a peptide monomer (Equation (6)). Further, for polysaccharides, $5l\leq L_{p}\leq 10l$; whereas, for polypeptides, $L_{p}\approx 5l$, and thus, the denominator term diminishes further in Equation (7) for polypeptides.

As highlighted earlier, the charge density of a polypeptide is lower when compared to a polysaccharide. Thus, the results are restricted to an f value of 0.25 (i.e., at most, one in every four amino acids are charged). Even at such low charge densities, it is observable that a polypeptide chain is likely to swell more rapidly when compared to a fully charged polysaccharide chain. However, it is worth bearing in mind that the size of the coil is still strongly dependent on the persistence length of the polymer in question, and thus, as shown in Figure 3, the polysaccharides still exhibit much larger intrinsic viscosity values when compared to polypeptides, such as gelatin.

### 5.2. Molar Mass Dependence

Traditionally, the determination of the intrinsic viscosity is of interest to calculate a polymer’s molar mass (M) using the Mark–Houwink equation [10]:

(8) $$\left[\eta \right]=KM^{a}$$

where K and a are experimentally derived variables that depend on the polymer–solvent interactions. This expression may then be suitably rewritten to calculate the relative intrinsic viscosity term (${\left[\eta \right]}_{R})$:

(9) $${\left[\eta \right]}_{R}={\left(\frac{M_{2}}{M_{1}}\right)}^{a}$$

Here, $M_{1}$ and $M_{2}$ represent two distinct values of molar mass. For multiple unscreened polysaccharide systems in good solvents, the value of a is reportedly around 1 [27,28]. However, in all other cases, the biopolymer chains are expectedly more flexible, and thus, 0.5 < a < 1 (in the good solvent limit) [29]. Thus, it is noticeable that, in the best-case scenario, Equation (9) becomes a linear relationship. In all other cases, it is a sublinear power law dependence. In contrast, it is observable from Equation (7) that screening-mediated changes in ${\left[\eta \right]}_{R}$ take on a superlinear power law dependence (${\left[\eta \right]}_{R}\approx {\left(1+\Theta_{p}\right)}^{3/2}$).

It is, therefore, worth highlighting that screening effects are considerably more influential in changing the overall hydrodynamic coil size compared to changes in the molar mass in the case of (charged) biopolymers. Further still, biopolymers of the same molar mass can show dramatic changes in their coil size due to charge-mediated swelling. This latter point is of relevance in the estimation of a polymer’s molar mass. As discussed earlier, the molar mass of biopolymers is typically represented using $M_{w}$. However, $M_{w}$ is dependent on the size of coils in solution and is, therefore, highly sensitive to both the pH and ionic strength of the system. As such, typical acid–base (or base–acid) precipitation methods [2,7] used to extract biopolymers impact both parameters and, thus, introduce considerable variability in the size and solubility of biopolymer coils. Additionally, it also brings into question the traditional approach of relating physical properties, such as the viscosity, to the molar mass [10,14]. The intention of relating properties to the molar mass is to highlight underlying mechanisms of chain relaxation [10,14]. However, it is equally accepted that there are multiple methods to determine the molar mass and that the various techniques respond differently to the change in the molar mass distribution [10]. An argument may yet be made that it is still possible to carefully assess the molar mass using multiple techniques for a particular polymer–solvent system. However, this approach fails in the case of biopolymers where the exact chemical structure is unknown and might be nearly impossible to establish [7]. In these cases, there is still the possibility to represent trends in viscosity using the intrinsic viscosity (or the size of the macromolecular entity) as the point of reference. This is also the subject of the authors’ interest and is discussed in an alternate publication [30].

## 6. Conclusions

To summarize, it is found that the OSF model is quite suitable to model screening-mediated variations in the hydrodynamic size of a generic biopolymer system. The approach, based on this model, is successful in providing an accurate prediction for a number of polysaccharide architectures, charge levels, and types of functionalization using a bare persistence length of 7 nm. This value is closely comparable with the recently reported range of persistence lengths for cellulosic backbones. Equally, as expected (both theoretically and experimentally), completely screened polyampholytic polypeptide chains show minimal changes in their intrinsic viscosity despite changes in the pH. However, it is recognized that this study is limited to a few polysaccharides and only one polypeptide. Thus, the inclusion of other (bio)polymers that are relevant for biomedical applications, food and agriculture, and potentially other industries can further establish the validity of the current approach. Extending this study to include purely cationic/anionic polypeptides and DNA, for instance, provides the scope to investigate a wider range of persistence lengths and charge levels due to a single type of charge (+ or −). In contrast, the inclusion of RNA allows for a similar comparison with ampholytic (and even zwitterionic) polypeptides such as gelatin. Certainly, such comparisons may also be facilitated using synthetic weak polyelectrolytes. In addition, the potential difficulties of estimating a biopolymer’s molar mass based on coil size are worth emphasizing, as this is somewhat prone to errors due to charge. Moreover, in case the precise molecular structure is unknown, the use of the size of the macromolecular object (or [η]) as a direct internal reference still allows for underlying trends in the physical properties of biopolymer systems to be established. Although the role of screening-mediated variations in the hydrodynamic size remains the primary focus of the current study, variations in “soft” interactions due to changes in the pH or temperature are equally relevant in the context of biopolymers, which, as such, also remain underdeveloped. It is, therefore, encouraged to extend the current approach to these cases to cover a wider range of structure–property relationships for not only biopolymers but also for other weak polyelectrolytes, which may help in developing the physical understanding of charged (bio)polymers overall.

## Figures and Tables

**Table 1:** List of polymers and their salient properties: type of functionalization, architecture, and molar mass ^1^.

Polymer Name	Salient Properties
Sodium Alginate (Na—Alginate), CAS Number: 9005-38-3	Polyanion: Carboxyl group, High mannuronic acid content. Linear copolymer.
Chitosan, CAS Number: 9012-76-4	Polycation: Amine group, Linear homopolymer, $M_{w}$ ~50 to 190 kg/mol.
Sodium Carboxy Methyl Cellulose (Na—CMC), CAS Number: 9004-32-4	Polyanion: Carboxyl group, Degree of Substitution = 0.9, Linearly substituted homopolymer. $M_{w}$ ~250 kg/mol.
Pectin from Citrus Peels (Pectin—Citrus), CAS Number: 9000-69-5	Polyanion: Carboxyl group, Galacturonic acid ≥ 74.0%, degree of methylation ≥ 6.7%, Branched heteropolymer.
Gelatin from Porcine Skin (Porcine—Gelatin), CAS Number: 9000-70-8	Polyampholyte: Carboxyl group and amine group, Linear—collagen derivative.

^1^ The (range of) molar masses reported here are the ones provided by the manufacturer. No additional measurements were performed to assess the accuracy of the quoted molar mass. None of the quoted values were required for the theoretical calculations presented in the main text.

**Table 2:** Experimentally obtained solvent viscosities.

Solvent	$\eta_{s}\;$ (mPa·s) ± S.D. ^1^
Water, 298 K—No Added Salt	0.871 ± 0.005
Water, 298 K—Added Salt	0.880 ± 0.006
Water, 338 K—Added Salt	0.489 ± 0.002

^1^ S.D. = standard deviation.

**Table 3:** Experimentally obtained values for the intrinsic viscosity.

Biopolymer System	$\left[\eta \right]$ (m^3^/kg) ± S.D. ^1^
Screened: Na-Alginate	0.308 ± 0.065
Screened: Chitosan	0.795 ± 0.100
Screened: Gelatin—Porcine, T = 338 K, pH = 6	0.102 ± 0.024
Screened: Na-CMC	1.034 ± 0.037
Screened: Pectin—Citrus	0.332 ± 0.064
Unscreened: Na-Alginate	1.183 ± 0.157
Unscreened: Chitosan	4.636 ± 0.379
Unscreened: Na-CMC	5.624 ± 0.626
Unscreened: Pectin—Citrus	1.152 ± 0.175
Screened: Gelatin—Porcine, T = 338 K, pH = 4	0.095 ± 0.017
Screened: Gelatin—Porcine, T = 338 K, pH = 11	0.060 ± 0.017

^1^ S.D. = standard deviation.

**Table 4:** Experimentally obtained values for ${\left[\eta \right]}_{R}$ vs. the range of theoretically calculated values of ${\left[\eta \right]}_{R}$ for polysaccharides. The column labelled f alongside the footer provides a brief description of the values that were chosen for the theoretical calculations.

**Biopolymer**	${\left[\eta \right]}_{R}$ Expt.	$\Theta_{p}$ Expt.	${\left[\eta \right]}_{R}\;$ Theory	$\Theta_{p}\;$ Theory	f
Sodium Alginate	3.84	1.45	3.13–5.93	1.14–2.28 ^1^	0.83
Chitosan	5.83	2.24	4.32–8.93	1.65–3.30 ^1^	1.00
Sodium Carboxy Methyl Cellulose	5.44	2.09	3.58–7.05	1.34–2.68 ^1^	0.90
Pectin from Citrus Peels	3.48	1.30	2.39–4.13	0.79–1.57 ^1^	0.69

^1^ Lower Bound: $L_{p}=$ 10 nm, Upper Bound: $L_{p}=$ 5 nm.

**Table 5:** Experimentally obtained values for ${\left[\eta \right]}_{R}$—gelatin at different pH values vs. theoretically obtained values for ${\left[\eta \right]}_{R}$—polypeptides at different f values.

Label	${\left[\eta \right]}_{R}$	$\Theta_{p}$
Experiment: Gelatin pH = 6/Gelatin pH = 4	1.07	0.05
Experiment: Gelatin pH = 6/Gelatin pH = 11	1.71	0.43
Theory: f= 0.05	1.25	0.16
Theory: f= 0.10	2.11	0.65
Theory: f= 0.25	11.3	4.05

## Data Availability

The original data presented in this study are openly available in the 4TU.ResearchData repository at https://doi.org/10.4121/f1ab8cce-67ec-4a8d-8f3b-4471db5d372e.

## Supplementary Materials

The following supporting information can be downloaded at: https://www.mdpi.com/article/10.3390/polym16202894/s1, Section S1: Conductivity Due to Counterion Condensation; Section S2: Scaling Approach.

## Notations

List of symbols in order of their appearance.

**Symbol**	**Meaning**	**Units**
η	Viscosity of solution	Pa·s
$\eta_{S}$	Viscosity of solvent	Pa·s
ϕ	Volume fraction	-
$\left[\eta \right]$	Intrinsic viscosity	m^3^/kg
c	Mass concentration	kg/m^3^
〈R〉	End-to-end distance	nm
$L_{c}$	Contour length	nm
$L_{p}$	Persistence length	nm
N	Number of repeating units	-
l	Monomer size	nm
$\langle R_{g}\rangle$	Radius of gyration	nm
M	Molar mass	kg or kg/mol
$\phi_{YF}$	Numerical function from Yamakawa—Fujii model	m^3/2^ or nm^3/2^
$M_{w}$	Weight-averaged molar mass	kg/mol
$L_{p}^{OSF}$	Electrostatic persistence length	nm
$L_{p}^{+}$	Persistence length of unscreened polymer chain	nm
$L_{B}$	Bjerrum length	nm
f	Fraction of charged monomers	-
κ	Inverse Debye length	nm^−1^
${\left[\eta \right]}_{U}$	Intrinsic viscosity of unscreened biopolymer	m^3^/kg
${\left[\eta \right]}_{S}$	Intrinsic viscosity of screened biopolymer	m^3^/kg
${\left[\eta \right]}_{R}$	Relative intrinsic viscosity	-
$\Theta_{P}$	Expansion factor	-
