# Supplementary material for: Charge Mediated Changes to the Intrinsic Viscosity of Biopolymer Systems
Source: Polymers (Basel). 2024 Oct 14;16(20):2894. doi: 10.3390/polym16202894 (PMC11511010; doi:10.3390/polym16202894)
Supplement: Supplementary file 1 [file polymers-16-02894-s001.zip › polymers-3244855-supplementary.pdf]

Supplementary Material for Publication

Charge Mediated Changes to the Intrinsic Viscosity of Biopolymer Systems

Authors and Affiliations

Anand Raja[a*], Philipp K. Wilfert[b,1], Stephen J. Picken[c]

a: Advanced Soft Matter, Department of Chemical Engineering, TNW – Applied Sciences, Delft University of Technology, van der Maasweg 9, 2629 HZ, Delft, The Netherlands. **Email**: A.Raja-1@tudelft.nl

b: Environmental Biotechnology, Department of Biotechnology, Delft University of Technology, TNW – Applied Sciences, Delft University of Technology, van der Maasweg 9, 2629 HZ, Delft, The Netherlands. **Email**: P.K.Wilfert@tudelft.nl

c: Advanced Soft Matter, Department of Chemical Engineering, TNW – Applied Sciences, Delft University of Technology, van der Maasweg 9, 2629 HZ, Delft, The Netherlands. **Email**: S.J.Picken@tudelft.nl

*: Corresponding Author

S1. Conductivity due to Counterion Condensation

We present here our calculations for three biopolymer systems for which the chemical structure remains well established: Na-Alginate, Na-CMC, and Chitosan. Although no specific information about the counterion is provided in the case of Chitosan, we assume it to be Cl^{-} by virtue of using a HCl solution to adjust the pH. Based on this information, we calculated the moles of counterion present per gram of polymer. This is summarized in Table S1.

As mentioned in our main text, we highlight a linear dependence between conductivity and concentration. We therefore estimate the conductivity at different polymer concentrations using the limiting molar conductivity values (at 25 °C) for Na^{+} and Cl^{-}, i.e., 0.005 S·m²/mol and 0.008 S·m²/mol respectively[1]. The results are depicted graphically in Figure S1.

In Figure S1, the estimated values of the conductivity are in good agreement with the measured values for Na-Alginate as well as Na-CMC. However, we observe that our estimations in the case of Chitosan overpredicts the expected conductivity. Further, based on the evidence provided in literature[2], the dependence on the concentration is not linear and we would in fact expect a reduction in the conductivity of the polymer in the concentrated regime. Based on these considerations, we suspect that our approach here merely provides an initial

estimation for the contribution made by the counterions, and that an investigation into this matter remains outside the scope of our current work.

Table S1: This table summarizes the calculations for the moles of counterion per gram of polymer.

Biopolymer	Mass of Counterion / Mass of Monomer	Moles of Counterion per kg of polymer
Na-Alginate	0.116	5.051
Na-CMC	0.088	3.846
Chitosan	0.180	5.091

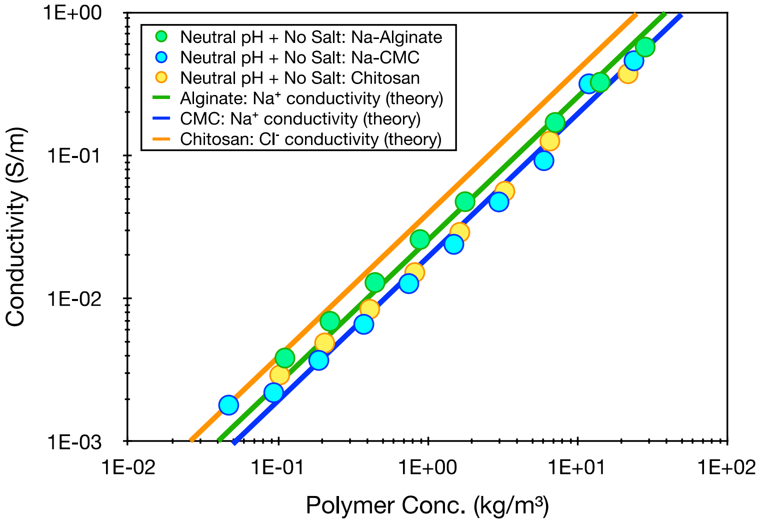

Figure S1: This figure represents both, the measured conductivity values of the "Neutral pH – No Salt" samples as well as the estimation of the counterion contributions (expressed in brackets as theory) to the electrical conductivity of the system.

S2. Scaling Approach

In the theoretical approach we have highlighted, we preserve the use of a semi-flexible "worm like" idealisation of biopolymer chains. Alternatively, Dobrynin, Colby & Rubinstein (DCR) propose the possibility to model polyelectrolytes using a scaling approach that utilises electrostatic blobs[3]. When dissolved in a good solvent, electrostatic interactions of each blob are of the order of the thermal energy. Therefore, the size of each electrostatic blob is governed by L_B. Further, as proposed by the DCR model, on length scales larger than the size of the blob, the electrostatic repulsions start to dominate and thus, the chain may be considered as a rodlike assembly of electrostatic blobs with length L (good solvent limit):

$$L = Nl\left(\frac{u}{A^2}\right)^{\frac{2}{7}} \quad \text{(S1)}$$

Where $u = L_B/l$ and A is the average number of monomers between charges. Thus, it is possible to approximate the radius of gyration for this rod like chain as being:

$$R_g = \frac{L}{\sqrt{12}} = \frac{Nl}{\sqrt{12}}\left(\frac{u}{A^2}\right)^{\frac{2}{7}} \tag{S2}$$

Similarly, Equations 2 and 3 (from main text) may be simplified further to yield an R_g in the screened limit (for a linear chain):

$$R_g = \sqrt{\frac{L_c L_p}{3}} = \sqrt{\frac{NP}{3}} \cdot l \tag{S3}$$

Where $P = L_p/l$. Thus, from Equations (S2) and (S3), it is possible to approximate $[\eta]_R$ as follows:

$$[\eta]_R \approx \frac{1}{8} \cdot \left(\frac{N}{P}\right)^{\frac{3}{2}} \cdot \left(\frac{u}{A^2}\right)^{\frac{6}{7}} \tag{S4}$$

It is worth noting that in Equation S4, we continue assuming an equivalence between the hydrodynamic radius and the radius of gyration, and do not necessarily overcome the limitations we addressed earlier (see Section 2). Additionally, as Equation S4 demands prior knowledge of the molar mass (through N), we limit our comparison here to the CMC system (Table S2).

Table S2: A comparison between experimental results, the worm-like chain approach, and the rod like chain approach for sodium carboxymethyl cellulose ($L_P = 7$nm, refer to Figure 4 in main text)

Approach	$[\eta]_R$
Experimental, CMC	5.441
OSF Approach	4.969
DCR Approach	57.21

It is clear from Table S2 that the rod like approximation provided by the DCR model is much larger when compared to both, the OSF approach as well as the experimental value. Thus, the scaling approximation of the polymer chains in the unscreened case may be a very coarse interpretation for the conformation (at least in the case of polysaccharides). The DCR approach highlights that $u/A^2 < 1$ typically, and thus suggests that there is still some flexibility on length scales smaller than the electrostatic blob size[3]. However, we believe that this approach is valid only when $A^2 \gg 1$. In the case of Chitosan $A^2 \approx 1.23$ and thus, although the DCR approach captures some flexibility within the polysaccharide chain, the chain is still modelled as an inherently stiff rod like object. In the case of polypeptides $A^2 \gg 1$ and thus, the scaling approach may yet be successful in capturing the flexibility within the charged chain.

References

(1) Robinson, R. A.; Stokes, R. H. *Electrolyte solutions*; Courier Corporation, **2002**.

(2) Bordi, F.; Colby, R.; Cametti, C.; De Lorenzo, L.; Gili, T. Electrical conductivity of polyelectrolyte solutions in the semidilute and concentrated regime: the role of counterion condensation. *The Journal of Physical Chemistry B* **2002**, *106* (27), 6887-6893.

(3) Dobrynin, A. V.; Colby, R. H.; Rubinstein, M. Scaling theory of polyelectrolyte solutions. *Macromolecules* **1995**, *28* (6), 1859-1871.
